# Tutorial on laser speckle rheology: technology, applications, and opportunities

**DOI:** 10.1117/1.JBO.25.5.050801

**Published:** 2020-05-01

**Authors:** Zeinab Hajjarian, Seemantini K. Nadkarni

**Affiliations:** Massachusetts General Hospital, Harvard Medical School, Wellman Center for Photomedicine, Boston, Massachusetts, United States

**Keywords:** cell biomechanics, optical elastography, laser speckle rheology, optical micromechanical imaging, tissue biomechanics

## Abstract

**Significance:** The onset of several diseases is frequently marked with anomalous mechanical alteration of the affected tissue at the intersection of cells and their microenvironment. Therefore, mapping the micromechanical attributes of the tissues could enhance our understanding of the etiology of human disease, improve the diagnosis, and help stratify therapies that target these mechanical aberrations.

**Aim:** We review the tremendous opportunities offered through using optics for imaging the micromechanical properties, at length scales inaccessible to other modalities, in both basic research and clinical medicine. We specifically focus on laser speckle rheology (LSR), a technology that quantifies the mechanical properties of tissues in a rapid, noncontact manner.

**Approach:** In LSR, the shear viscoelastic modulus is measured from the time-variant speckle intensity fluctuations reflected off the tissue. The LSR technology is engineered and configured into several embodiments, including bench-top optical systems, endoscopes for minimally invasive procedures, portable point-of-care devices, and microscopes.

**Results:** These technological nuances have primed the LSR for widespread applications in diagnosis and therapeutic monitoring, as demonstrated here, in cardiovascular disease, coagulation disorders, and tumor malignancies.

**Conclusion:** The fast-paced technological advancements, elaborated here, position the LSR as a competent candidate for many more exciting opportunities in basic research and medicine.

## 1 Quantifying Tissue Mechanics

The pathogenesis and progression of disease is frequently associated with serial changes in the mechanical properties of multiple functional units from organs and tissues to the extracellular matrix (ECM), cells, and subcellular structures.^1^^–^^3^ Biological tissues are viscoelastic, exhibiting both solid-like (elastic) and liquid-like (viscous) behaviors, under different loading conditions. This mechanical behavior is best quantified by the frequency-dependent shear viscoelastic modulus, $G^{*}(\omega )=G^{\prime }(\omega )+iG^{\prime }(\omega )$.^4^ Here, the real part, $G^{\prime }(\omega )$ is the elastic or storage modulus, a measure of solid-like behavior of the sample. On the other hand, the imaginary part, $G^{\prime \prime }(\omega )$ is the viscous or loss modulus and is a measure of viscous energy dissipation by the sample.^4^ Shear modulus varies markedly among biological tissues in different organs and is closely linked to the tissue function.^1^^,^^5^^,^^6^ This metric puts a number on the qualitative feeling of hardness, assessed by clinicians via physical palpation.^5^ Mechanical rheometry is the standard approach for evaluating the shear modulus, in which the specimen is subjected to an oscillatory shearing between two plates, and the ratio of applied stress to the resulting strain is measured over a limited oscillation frequency range.

In biological science and medicine, several qualitative and quantitative techniques are exploited to assess the mechanical properties of tissues at different length scales for both basic science and clinical applications.^6^ On the clinical side, noninvasive elastography modalities have emerged based on the conventional ultrasound and magnetic resonance imaging, which measure the deformation in response to mechanical loading and map the local mechanical properties of the tissue. These modalities are regarded as the image-based equivalents to palpation, in deep fields, inaccessible to touch, except for during surgery.^5^^,^^7^^–^^9^ The advent of these modalities has tremendously improved the diagnosis in liver, thyroid, breast, prostate, kidney, and lymph node pathologies through exploiting the mechanical contrast between healthy and pathological states.^9^ Two general classes are available, namely strain and shear-wave elastography, with the former based on evaluating the deformation in response to the internal or external compression loads, and the later based on tracking the stimulated traveling shear waves to assess elasticity.^5^^,^^7^^–^^9^ These conventional elastography techniques evaluate the mechanical properties of tissues at the whole-organ scale, with mm-cm resolutions.^10^ Nevertheless, new insights emerging in the fields of cellular biomechanics and mechanobiology emphasize the intimate link between micromechanical alterations at cellular scales and the emergence and progression of pathological conditions.^1^^,^^11^^,^^12^ They further stress the need for new tools that enable noninvasive mapping of micromechanical attributes, such as the viscoelastic modulus, force, and stress to understand the etiology of the disease, to improve the diagnosis, and to design therapies that target the mechanical aberrations.^13^^,^^14^

## 2 Optical Imaging of Tissue and Cell Mechanics

In the past decade, several optical techniques have emerged to probe the biomechanical properties at cellular scales.^13^^,^^15^^,^^16^ Active optical mechanical sensing methods often entail physical contact with the specimen to apply external loading and induce deformation. Major examples include different varieties of optical coherence elastography (OCE) which exploit the superior resolution of the optical coherence tomography (OCT) to map the deformation in response to the static or dynamic external loads.^10^^,^^17^^,^^18^ Compression OCE uses a static compressive load, applied to the tissue surface, for instance by a piezo electric transducer.^19^ The gradient of displacement with depth is then measured as the local strain and presented as an elastogram. The assumption of a uniform stress throughout the tissue volume enables calculating the elastic modulus as the ratio of the stress to strain. This assumption remains valid in homogeneous or layered specimens but is violated in heterogeneous specimens, containing inclusions.^10^ While the lateral resolution of compression OCE remains the same as the primary OCT system, the axial resolution is reduced, 5 to 10 times, to ∼100  μm due to need for calculating the gradient of displacement over several pixels along the depth.^17^ Compression OCE has been demonstrated to enable tumor margin detection, based on the drastically increased micromechanical heterogeneity of the tumor compared to the mature stroma in adjacent normal tissue.^20^ The alternative transient varieties of OCE involve the use of actuators, ultrasound, or laser-generated acoustic radiation forces, or air puffs to create acoustic waves.^21^^–^^23^ Subsequently, OCT is used to track either of the surface, shear, or Lamb waves that are generated depending on the depth of excitation and tissue structure. While the exact relationship may vary, the phase velocity of acoustic waves is generally proportionate to the square root of elastic modulus.^17^^,^^18^^,^^21^^–^^23^ The lateral resolution of transient OCE is set by the speed of acoustic waves and the frame rate of the parent OCT system to a few hundreds of μm.^17^^,^^18^ In principle, the lateral resolution may be increased to a few tens of μm by repeated mechanical loading and obtaining multiple M-mode scans at various locations. Nevertheless, this would significantly increase the acquisition time.^24^ The axial (depth) resolution of these OCE methods is limited, in principle, to the axial resolution of the underlying OCT system to ∼10  μm. Nevertheless, practical considerations may further reduce the depth resolution.^18^ Transient OCE modalities have been widely used in various soft and rigid tissues yet are particularly appealing in the context of ocular mechanics, within the nearly transparent anterior segment of the eye, containing cornea and crystalline lens.^10^^,^^18^^,^^21^^,^^25^ Among transient OCE modalities, the shear wave imaging-OCE based on an air-puff excitation demonstrates a direct clinical applicability for evaluating the mechanical properties of human cornea in a clinical settings.^18^^,^^21^^,^^25^

Endogenous motions may also be used as a source of deformation, enabling all-optical, noncontact mapping of the viscoelastic properties within the biological tissue, such as in laser speckle rheology (LSR) and Brillouin microscopy.^26^^–^^46^ Brillouin scattering refers to an inelastic scattering caused by the interaction of light with intrinsic mechanical vibrations (spontaneous acoustic phonons) within the material.^47^ The frequency shift of the Brillouin scattered light is proportionate to the velocity of the local spontaneous acoustic waves, in turn proportional to the square root of the high-frequency longitudinal modulus.^26^^,^^48^ The longitudinal modulus is proportionate to the elastic modulus, although the coefficient of proportionality is not constant and may depend on the specific tissue or cell type. By employing a confocal geometry, with a high numerical aperture objective, and a parallel spectrometer, Brillouin microscopy achieves superb axial and lateral resolutions (1.5 and 0.3  μm), with demonstrated application at the *in-vivo* tissue and *in-vitro* cell levels.^27^^–^^29^^,^^49^^–^^51^

Use of optics is not limited to measuring the viscoelastic properties in tissues; it also extends to evaluating the force and stress exchanged during cell–ECM biomechanical interactions, which are also indirectly tied back to the mechanical properties of tissue scaffoldings. More specifically cells sense the stiffness of their substrates through transmembrane integrins and respond by contractile forces at the sites of focal adhesions. Traction force microscopy (TFM) exploits the motion of fluorescent fiduciary beads to evaluate these forces at the sites of focal adhesion assemblies, to investigate the cross-talk between the mechanical cues conferred by the ECM, the subsequent activation of cellular mechanosignaling, and the hallmarks of malignant transformation.^52^^–^^57^ Despite efforts toward combining TFM with confocal microscopy, tracking vertical displacements and the corresponding small perpendicular forces remains challenging in TFM.^58^ In addition, this technique is associated with phototoxic effects and does not yield for either longitudinal studies or immunohistochemical analysis of the cells following the completion of the experiments.^59^^,^^60^ More recently, a sophisticated elastic resonator interference stress microscopy (ERISM) has been proposed to overcome these limitations.^59^^,^^60^ In ERISM, cells are seeded on top of an optical microcavity and their vertically applied forces are measured from the local thickness of the cavity, calculated my mapping the reflectance spectra across the bottom surface. Minute vertical forces have been evaluated with ERISM, including those involved in podosome protrusion, protein-specific cell–substrate interaction, and amoeboid migration.^59^^,^^60^

## 3 Laser Speckle Rheology: Technology Platform

**Fig. 1:**
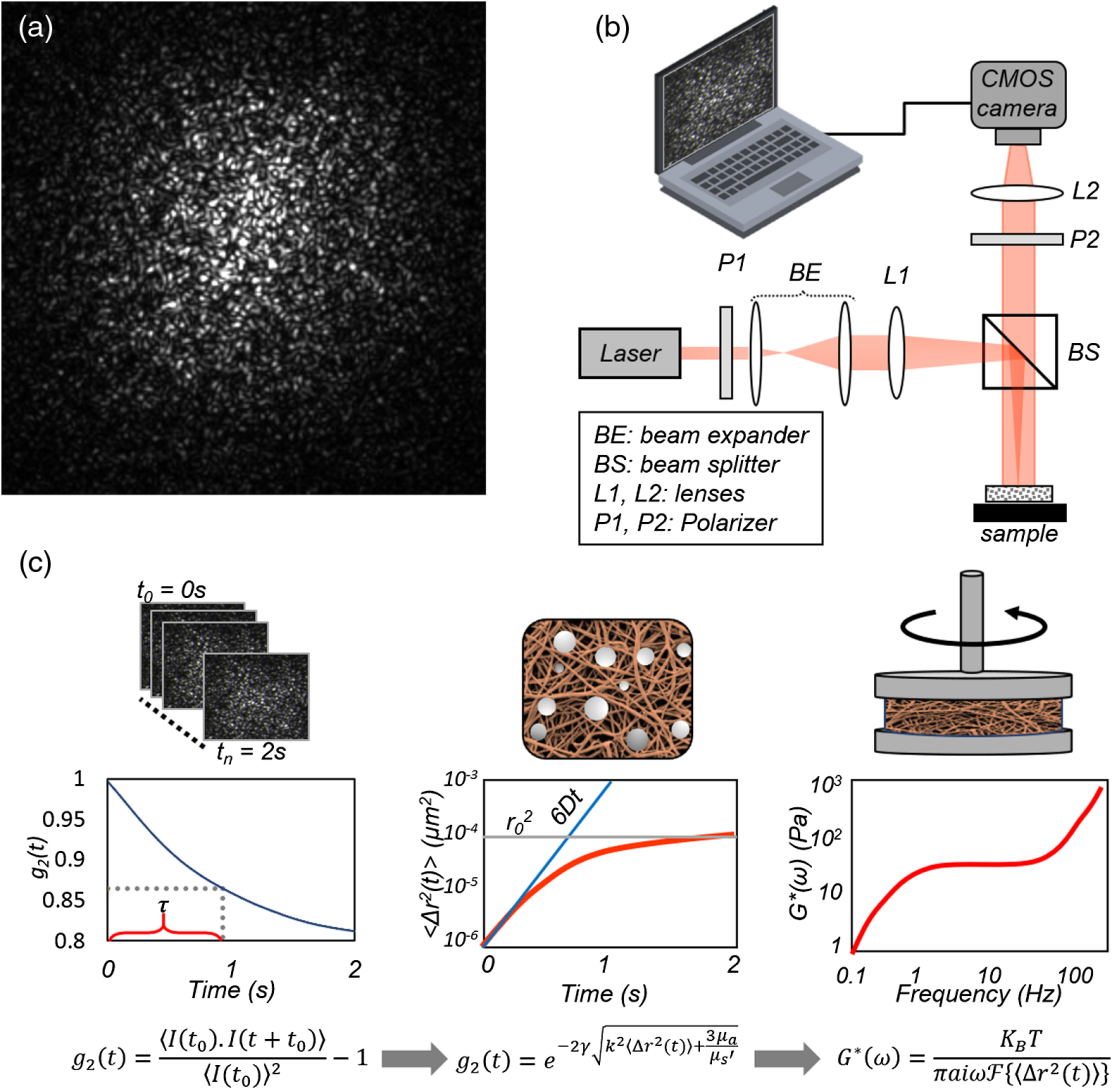
(a) A fully developed laser speckle pattern. (b) Schematic of the LSR optical layout. In short, light from a randomly polarized He–Ne laser (632 nm, 30 mW) is polarized, collimated, using a beam expander, bent, and brought to focus by a beam-splitter (BS) and a lens ($L_{1}$, focal length 25 cm, 50-mm spot size) at the sample surface. The cross- and co-polarized components of backscattered light are captured by a CMOS high-speed camera, equipped with a focusing lens system. The acquired speckle frame series are transferred to a high-speed computer for further processing. (c) Theoretical principles of the LSR processing scheme used to measure the shear viscoelastic modulus from the speckle intensity fluctuations.^30^^,^^31^^,^^34^^–^^38^^,^^40^^,^^41^

LSR is an optical technique that evaluates the viscoelastic properties of biological tissues and biomaterials, without applying an external force, physically contacting the specimen, or using exogenous particles, in a passive, noninvasive approach.^30^^–^^35^^,^^37^^–^^46^^,^^61^^–^^64^ Laser speckle [Fig. 1(a)] is a random granular pattern of dark and bright spots that emerges when a highly coherent beam of light, e.g., laser, is backscattered from a particulate material, such as the biological tissue.^65^ Continuous thermal movements of endogenous scattering particles modify the optical phase shifts of backscattered rays and lead to time-varying speckle intensity fluctuation. These fluctuations are highly sensitive to particle displacements, and in-turn to the viscoelastic properties of the microenvironment surrounding the particles.^30^^–^^35^^,^^37^^–^^46^^,^^64^ The optical layout of a bench-top LSR system is shown in Fig. 1(b).^30^^,^^31^^,^^34^^–^^38^^,^^40^^,^^41^ Briefly, light from a laser source of long coherence length is polarized and focused at the luminal surface of the sample. Light rays penetrate the sample and scatter multiple times before emerging back to the surface. The backscattered light is passed through an analyzer and projected by an imaging lens on to the sensor of a high-speed CMOS camera. The interference of light rays gives rise to the speckle pattern at the camera sensor.^30^^,^^31^^,^^34^^–^^38^^,^^40^^,^^41^ The acquisition frame rate of the camera is adjusted according to the speckle dynamics, between a few hundreds to thousands of frames per second (fps), such that the individual speckle frames are fully developed, exhibiting dark and bright spots of maximum contrast. This is realized when the speckle optical field amplitude follows a zero-mean, Gaussian distribution, creating an exponentially distributed speckle intensity of equal mean, and standard deviation. On the other hand, if the frame rate is smaller than the time scales of Brownian motion dynamics, multiple speckle patterns that evolve in time, are acquired during a single camera exposure duration, creating blurred and smeared-out speckle spots. The resulting optical field with a nonvanishing mean induces a steady, non-zero mean intensity level, and a lower speckle contrast.^34^^,^^35^^,^^41^^,^^66^^–^^68^

Speckle contrast is also impacted when the speckle grain size is smaller than the size of at least 2 pixels, causing multiple speckle spots to overlap in space, average out, and create a steady gray background in the speckle image.^34^^,^^35^^,^^41^^,^^66^^–^^68^ The speckle frames are transferred to a high-speed computer, where multiple processing steps are followed to calculate the viscoelastic properties. Theoretical principles of LSR are shown in Fig. 1(c). Typically, the speckle intensity temporal autocorrelation curve, $g_{2}(t)$, is obtained by measuring the correlation between pixel intensities in the first speckle frame with subsequent frames, over the imaging duration.^30^^,^^31^^,^^34^^–^^38^^,^^40^^,^^41^ To evaluate the bulk modulus, spatial averaging is performed over all the pixels in the frame, and several $g_{2}(t)$ curves evolving in time are averaged to enhance the accuracy of temporal statistics, as follows:^30^^,^^31^^,^^34^^–^^38^^,^^40^^,^^41^^,^^69^

$$g_{2}(t)=\frac{\left\langle I(t_{0})I(t+t_{0})\right\rangle }{{\left\langle I(t_{0})\right\rangle }^{2}}.$$ (1)

From this curve, the mean square displacements (MSD), i.e., $\left\langle \Delta r^{2}(t)\right\rangle$, of scattering particle, may be retrieved. In low viscosity liquids, rapid diffusive movements of scattering particles induce a linear trend for MSD that grows quickly with time, eliciting rapid speckle fluctuations, and $g_{2}(t)$ decorrelation. On the other hand, in highly elastic solids, the sluggish local vibrations of scattering particles are represented by a saturated MSD and indolent speckle variations.^34^^,^^40^^,^^42^ Acquiring low contrast speckle frames could slow down the $g_{2}(t)$ decorrelation and artificially increase the $g_{2}(t)$ plateau level at longer times.^34^^,^^35^^,^^41^^,^^66^^,^^67^ By obscuring the sample dynamics and masking the actual viscoelastic behavior at longer decay times, a low-contrast speckle acquisition could therefore reduce the dynamic range of LSR measurements.^34^^,^^35^^,^^41^ Careful selection of detector specifications is required to achieve optimum speckle contrast and accomplish LSR measurements. Early microrheology studies by Mason and Weitz demonstrated that the intrinsic thermal Brownian fluctuations, probed by MSD, closely represent the viscoelastic response of the medium to an external shear force, through the generalized Stokes–Einstein relation (GSER) as^70^^–^^74^

$$G^{*}(\omega )={\frac{K_{b}T}{\pi a\Gamma [1+\alpha (t)]\Delta r^{2}(t)}|}_{t=\frac{1}{\omega }}.$$ (2)

where $K_{B}$ stands for the Boltzman constant ($1.38\times 10^{-23}$), T is the temperature (degrees Kelvin), a represents the scattering particle radius, ω is the oscillation frequency, α is the logarithmic slope of the MSD, i.e., $=\frac{\partial \;\log\langle \Delta r^{2}(t)\rangle }{\partial \;\log(t)}$, and Γ is the gamma function.^70^^–^^74^ In these past studies, MSD was derived through tracking of sparsely dispersed exogenous beads in otherwise transparent gels using either video microscopy or dynamic light scattering (DLS). These approaches were not conducive for rapid, high throughput mapping of mechanical properties in biological tissues. Later on, development of the diffusing wave spectroscopy (DWS) formalism enabled extracting the MSD from the intensity fluctuations of the light scattered from very opaque, highly multiple-scattering media, where light propagation is nearly diffusive.^71^ Nevertheless, DLS and DWS only cover the two extremes of the wide range of optical properties spanned by the tissues and biomaterials.

Due to difficulties in accurate deduction of MSD and then $G^{*}$ from $g_{2}(t)$, early LSR studies extracted an index of viscoelasticity from the speckle fluctuations.^30^^,^^31^^,^^33^^,^^34^ This was achieved by fitting a single exponential function to $g_{2}(t)$ and obtaining the time-constant, τ, of the exponential decay, or the speckle decorrelation time.^30^^–^^34^ The first LSR study demonstrated the relationship between this viscoelastic index and the composition of the atherosclerotic plaques.^30^ Later studies demonstrated a strong, statistically significant correlation between the τ and the $G^{*}(\omega )$ evaluated by a rotational rheometer, over a large range of modulus values in phantom (R=0.79; p<0.0001) and tissue (R=0.88, p<0.0001) samples for $\left|G^{*}\right|$ between 60 and 600 kPa.^34^ Nevertheless, extracting the $G^{*}(\omega )$ from the speckle signal remitted from a tissue of unknown optical properties and scattering particle size presented a couple of challenges.^35^^,^^37^^,^^40^^,^^42^

The first challenge was related to isolating the influence of optical absorption and scattering, when evaluating the MSD from the measured $g_{2}(t)$ curve.^35^^,^^37^ This was because speckle fluctuations are driven by both Brownian displacement of scattering centers and the number of scattering events involved in photon trajectories. The photon transport within the illuminated volume is in turn determined by the optical absorption and reduced scattering coefficients ($\mu_{a}$, ${\mu_{s}}^{\prime }$).^35^^,^^37^ Therefore, to obtain the MSD from $g_{2}(t)$, one needs to quantify and compensate for $\mu_{a}$ and ${\mu_{s}}^{\prime }$. Previous efforts yielded the following analytical form based on the DWS formalism for $g_{2}(t)$ in the presence of optical absorption:^71^

$$g_{2}(t)=e^{-2\gamma \sqrt{k^{2}\left\langle \Delta r^{2}(t)\right\rangle +\frac{3\mu_{a}}{{\mu_{s}}^{\prime }}},}$$ (3)

where γ is an experimental parameter, equivalent to the normalized distance that light penetrates the sample before its propagation becomes completely diffusive. Thus, it is related to the contribution of short, nondiffusive optical paths, and may vary depending on the size of scattering particles and the effects of polarization.^71^ Moreover, k is the wavenumber. The additional term in the exponent accounts for the truncation of long optical paths due to absorption, which rounds off the initial decay of the $g_{2}(t)$ curve.^71^^,^^74^ Nevertheless, this equation is not valid for tissues of negligible $\mu_{a}$.^35^ To address this issue, we exploited a correlation transfer Monte–Carlo algorithm to simulate the photon propagation in moderately scattering materials that fall between the single scattering and diffuse-rich scattering limits. We then developed a modified equation to derive the MSD, from $g_{2}(t)$ in moderately scattering samples of negligible $\mu_{a}$, as^35^

$$g_{2}(t)=e^{-2\gamma {\left[k^{2}\langle \Delta r^{2}(t)\rangle \right]}^{\varsigma }}.$$ (4)

**Fig. 2:**
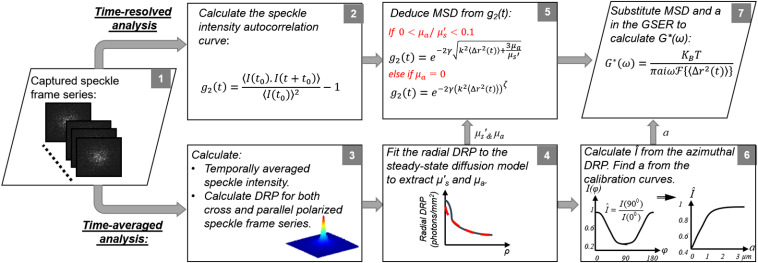
Flowchart of LSR processing algorithm (adapted with modifications from Refs. 35 and 42). Box 1: speckle images are acquired at both cross- and co-polarized states, with respect to linear polarization of the illumination beam. Box 2: cross-correlating the first speckle frame with subsequent frames provides the speckle intensity autocorrelation function, $g_{2}(t)$. Box 3: temporal averaging of speckle frames returns the DRP at both cross- and co-polarized states. Box 4: the $\mu_{a}$ and ${\mu_{s}}^{\prime }$ are experimentally evaluated via curve-fitting to the radial cross-polarized DRP. Box 5: optical properties determine whether the DWS formalism, i.e., $g_{2}(t)=\exp-2\gamma \surd [k^{2}\langle \Delta r^{2}(t)\rangle +3\mu_{a}/{\mu_{s}}^{\prime }]$ or the modified MCRT-driven equation, i.e., $g_{2}(t)=\exp-2\gamma {\left[k^{2}\langle \Delta r^{2}(t)\rangle \right]}^{\zeta }$, should be used to deduce the MSD. Box 6: the ratio of co-polarized DRP along short and long axes, i.e., I^=I(φ=90  deg)/I(φ=0  deg), is compared with a calibration curve to evaluate the average radius of scattering particles, a. Box 7: MSD and a are substituted in GSER to calculate the $G^{*}(\omega )$.

Here, γ and ζ are experimental parameter, related to ${\mu_{s}}^{\prime }$. We further proposed an approach to estimate $\mu_{a}$ and ${\mu_{s}}^{\prime }$, by temporally averaging the speckle frame series and obtaining the diffuse reflectance profile (DRP) of the specimen. We then fitted a model function, obtained from light diffusion approximation to the radial profile of DRP to calculate both $\mu_{a}$ and ${\mu_{s}}^{\prime }$.^41^ The second challenge in calculating the $G^{*}$ from MSD lied in Eq. (2), which requires *a priori* knowledge of the average, sphere-equivalent radius of the scattering particles.^40^ This is because in addition to optical properties, size of scattering particles also modifies the speckle fluctuations.^40^ From Eq. (2), it is noted that for a medium of given $G^{*}$, MSD depends on scattering particle size. To address this challenge, we demonstrated that when plotted with respect to azimuth angle, the parallel-polarized DRP, derived from time-averaged speckle frames, exhibits a trend related to average scattering size, a.^40^^,^^42^ In other words, as the scattering particle size increases, the DRP evolves from a bilobular, elliptical form, to a quadrifolium, clover-like pattern.^40^ We confirmed this phenomenon experimentally, in microbeads of different sizes, computationally, using polarized MCRT, and theoretically, using double-scattering formalism obtained from the Muller matrix.^40^^,^^42^ To implement this approach in LSR, co-polarized speckle images were temporally averaged and converted to relative DRP as a function of azimuth-angle. Next, the ratio of DRP across short and long axes, i.e., I^=I(ϕ=90  deg)/I(ϕ=0  deg), was calculated and compared with a calibration curve to identify the scattering particle size.^40^^,^^42^ Figure 2 summarizes the details of the LSR-processing algorithm.^35^^,^^42^ The demonstrated capability of LSR in providing an accurate and highly sensitive measure of the viscoelastic modulus in a noncontact and noninvasive manner has opened several powerful opportunities for developing numerous basic research and translational tools for several pathological conditions, as detailed in the next sections.

## 4 Applications of LSR in Biomedicine

### 4.1 Endoscopic LSR

**Fig. 3:**
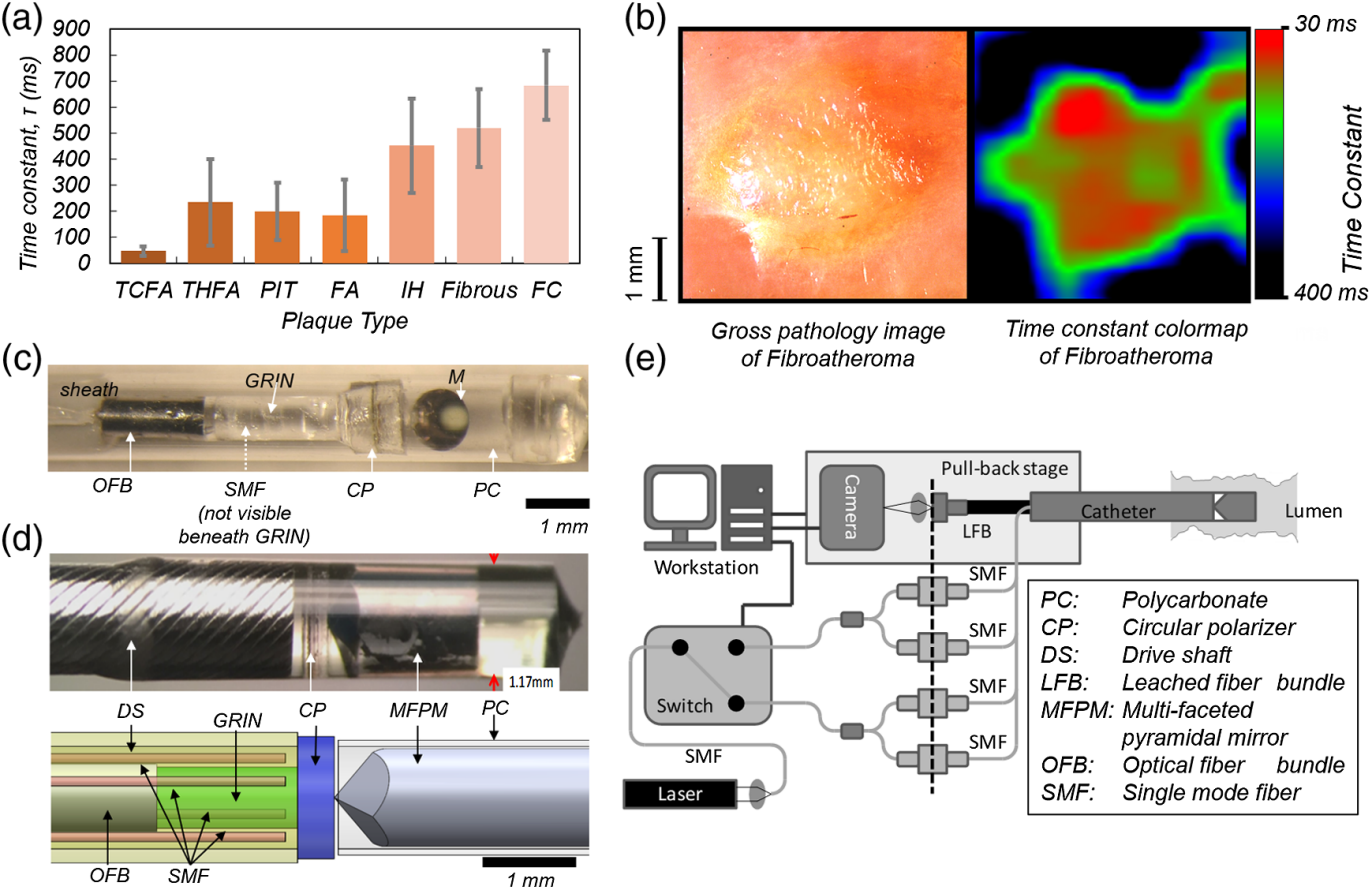
(a) Mean τ computed for different plaque groups under static conditions. The error bars indicate standard error of the mean. TCFA, thick-cap FA (THFA), pathological intimal thickening (PIT), nonnecrotic fibroatheroma (FA), intimal hyperplasia (IH), fibrous plaque, and fibrocalcific plaque (FC). (b) Color-map of the τ distribution (30 to 400 ms) over 4.5×4.5-mm ROI across lesion. Presence of lipid-rich plaque with a well-defined outline is clear from the color-map and is backed-up by the corresponding gross pathology photograph (adapted with modifications from Ref. 30). (c) The first-generation LSR endoscope. Reflection of the OFB is seen in the circular flat mirror, and the single-mode illumination (SMF) fiber runs parallel underneath the optical assembly (not visible). Scale bars: 1 mm (adapted with modifications from Ref. 33). (d) The omnidirectional LSR catheter assembly. Distal optics design is optimized for a lumen of 3 mm diameter (size of a human coronary artery). Top: The photograph of the distal optic assembly. The catheter diameter is 1.2 mm. Bottom: The computer-aided drawing shows the catheter distal optics assembly which incorporates SMFs for illumination, a circular polarizer (CP), an MFPM, a gradient-index (GRIN) lens, and an OFB. The distal assembly is housed within a protective polycarbonate (PC) tube and a customized drive shaft (DS). (e) Schematic diagram of the omnidirectional LSR catheter, pull-back assembly, and console hardware. Laser light (633 nm, 22.5 mW) is coupled into an SMF passes through an MEMS switch and is split to four illumination fibers. The speckle patterns obtained from the lumen wall are transmitted through an OFB and are imaged on the CMOS camera [(d) and (e) are adapted with modifications from Ref. 45].

Mechanical factors play a prominent role in the pathogenesis of several conditions in luminal organs, including coronary and peripheral arteries and gastrointestinal tract.^75^^–^^78^ For instance, acute myocardial infarction (AMI), the leading cause of death worldwide, is frequently caused by the rupture of mechanically unstable atherosclerotic plaques.^76^^,^^78^ In addition, gastrointestinal neoplasia is also associated with mechanical remodeling of the mucosa.^75^ Miniature LSR endoscopes and catheters enable delivering light beyond the tissue surface to inaccessible internal organs in a minimally invasive manner to evaluate the mechanical properties for improved diagnostic sensitivity and specificity. The motivation for endoscopic LSR raised following the seminal work by our group on *ex-vivo* arteries. These studies demonstrated that the speckle decorrelation time, τ, provides an index of viscoelasticity that is significantly different among plaques of different diagnosis and further identifies vulnerable thin cap fibroatheroma (TCFA), with a sensitivity of 100% and a specificity of 93% [Fig. 3(a)].^30^ They also demonstrated that depending on the illumination geometry, spatiotemporal analysis of speckle fluctuations could yield both a transverse two-dimensional (2-D) map of plaque stiffness [Fig. 3(b)] and a depth-resolved profile of stiffness to calculate the fibrous cap thickness.^31^ Compelling evidence on the detection and diagnostic yield of LSR launched feasibility studies that showed LSR can be conducted through small-diameter optical fiber bundles (OFBs) and highlighted the opportunity for endoscopic imaging through miniaturized catheters.^32^ Additional optimization of the low-cross-talk OFB design, with reduced core size and increased core-to-core spacing (>7  μm), enabled robust transmission of speckle patterns during physiological motions.^39^

These developments led to the fabrication of the first-generation endoscopic LSR prototype [Fig. 3(c)], a miniaturized side-viewing catheter (1.5 mm diameter) for the intraluminal assessment of tissue viscoelasticity.^33^ The catheter was interfaced with a portable console, incorporating the laser, the optical setup to couple light into the catheter, a high-speed CMOS camera for acquiring speckle patterns delivered by the fiber bundle, and a computer equipped with a frame grabber and data acquisition card.^33^ Intravascular testing in the aortas of rabbit models of atherosclerosis demonstrated the capability of intracoronary LSR for mechanical characterization of the arterial wall in living rabbits under the conditions of cardiac motion and respiration.^33^ This was followed by additional studies in human–swine xenograft models in conjunction with intracoronary balloon occlusion and saline flushing. The diagnostic capability of this side-viewing prototype catheter was limited, as it was only evaluating the speckle fluctuation at discrete spots along the wall, without mapping the circumference of the coronary vessel lumen.^33^^,^^34^ Circumferential viewing that is conventionally achieved by mechanically rotating the side-viewing catheters, such as for instance in OCT,^79^ did not present an appealing design for LSR. This was because the mechanical rotation of OFB could increase the coupling between multiple propagating modes with thousands of optical cores that transmit the speckle signal from the lumen wall to the camera and unpredictably modulate the speckle fluctuations. To overcome this limitation, our group proposed a design for the omnidirectional viewing LSR catheter for mapping the mechanical properties of luminal organs without the need for rotational motion, as shown in Figs. 3(d) and 3(e).^45^ This design featured multiple single-mode fibers (SMF), a customized multifaceted pyramidal mirror (MFPM) to guide light to various circumferential locations on the lumen wall, and an OFB to simultaneously collect multiple laser speckle patterns.^45^

By retracting the catheter using a motor-drive assembly, cylindrical maps of tissue mechanical properties are reconstructed without the need for mechanical rotation. The accuracy of the new omnidirectional LSR catheter measurements was confirmed in gel phantoms and human coronary arteries, using conventional mechanical rheometry as a reference standard.^45^ The rigorous development and preclinical testing in animal models, outlined above, pave the path for translating the LSR endoscopy systems to clinical setting, opening existing avenues for directly evaluating the viscoelastic properties of luminal organ in deep, inaccessible, uncharted fields.

### 4.2 *In-Vitro* Diagnostics

**Fig. 4:**
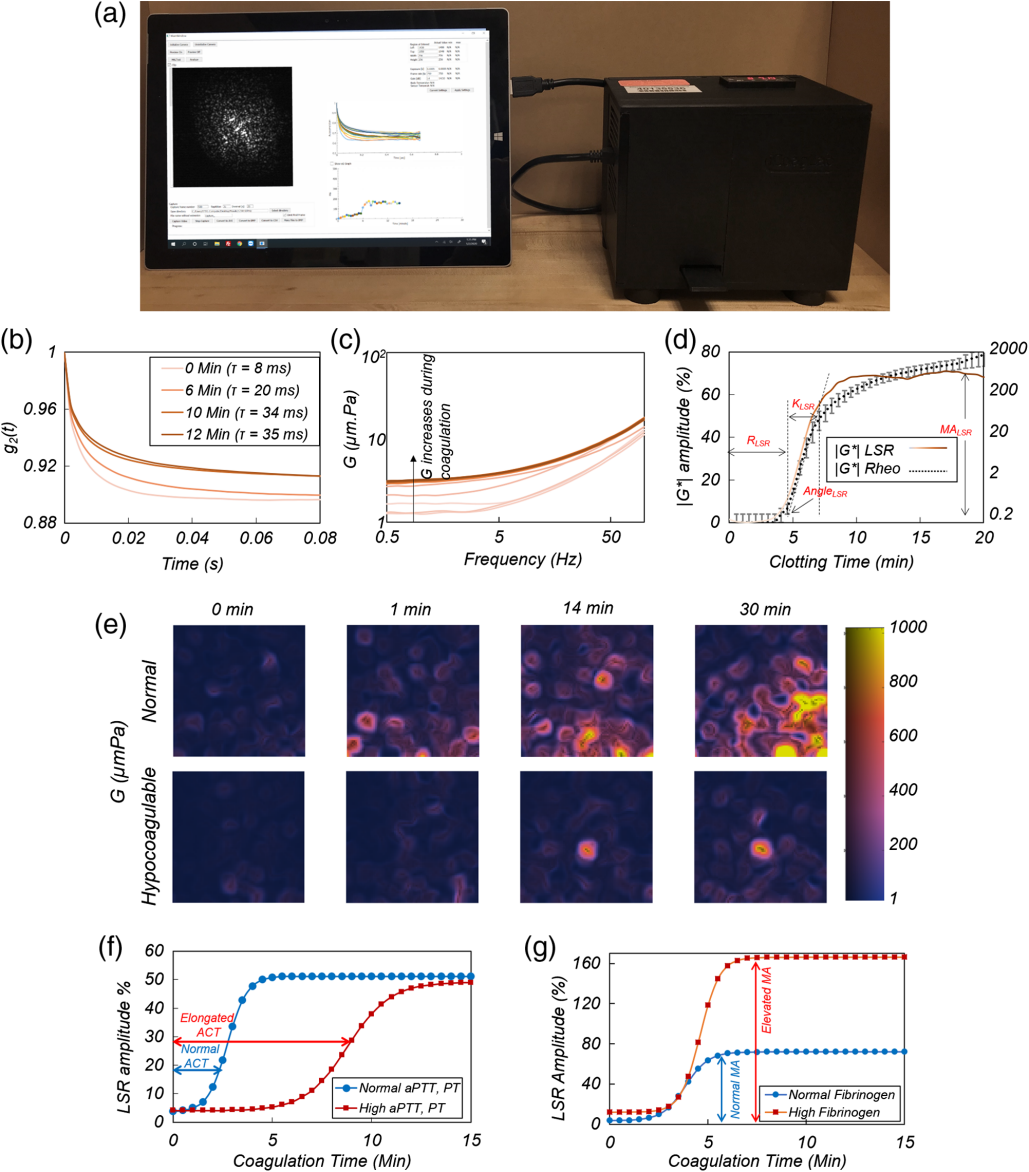
(a) Handheld LSR device (adapted with modifications from Ref. 43). (b) Temporal intensity autocorrelation curves measured from a blood sample during coagulation. The curves slow down as the blood clot forms, as appreciated from the increase in the speckle decorrelation time constant (adapted with modification from Ref. 38). (c) Frequency-dependent shear viscoelastic modulus, $G^{*}(\omega )$, measured by LSR increases during coagulation. (d) A typical LSR amplitude curve obtained by plotting the G at a specific frequency for normal blood, along with the $\left|G^{*}\right|$ trend measured using a rheometer, displayed on the secondary Y axis. The representation of the parameters, R, K, α angle, and MA, is displayed. (e) Spatial variation in G visualized in a drop of blood showing emerging initial microclots that form early in the coagulation process, the hypocoagulable sample shows negligible clotting (adapted with modifications from Ref. 41). Scale bars are 100  μm. (f) LSR amplitude curves for a patient with prolonged aPTT and PT. (g) LSR amplitude curves for a patient with elevated fibrinogen, compared with normal controls (adapted with modification from Ref. 51).

The LSR technology platform enables exciting avenues for simultaneous optical, biomechanical, and cytological analysis of biological fluids, using minutes volumes of the samples, in a noncontact and nonmanipulative approach.^35^^,^^38^^,^^40^^,^^41^ These multifunctional capabilities present tremendous opportunities for *in-vitro* diagnostics of impaired blood coagulation or coagulopathy, which is the leading cause of in-hospital preventable deaths.^80^^,^^81^ Management of coagulopathies requires rapid monitoring of the coagulation status at the point-of-care to prevent severe bleeding or for detecting and treating the potential thromboembolic conditions. Conventional coagulation tests (CCTs) measure the activated thromboplastin time (aPTT), prothrombin time (PT), fibrinogen levels, and clotting factors, along with platelet function to assess a patient’s coagulation status. Nevertheless, due to their long reporting times (1 to 5 h), CCT’s only provide a static snapshot of the coagulation status with a limited utility for dynamically changing coagulation conditions in surgical or trauma patients. Temporal evolution of blood viscoelasticity during coagulation reflects the time course of several biochemical processes in the coagulation cascade, including fibrin polymerization, clot stabilization, platelet–fibrin interaction, and fibrinolysis, and presents a direct indicator of blood coagulation status. This has motivated the development of rheology-based coagulation monitoring devices, such as thromboelastography (TEG^®^). Nevertheless, these tools entail moving mechanical components are sophisticated and demand routine calibration, which limit their widespread clinical utility.^82^ Moreover, contact-based mechanical testing is likely to strain the blood beyond the linear viscoelastic regime, prolong clot formation, and amend fibrin mesh structure, likely compromising the reliability of measurements in softer clots.^83^ Our lab has developed an LSR-based sensor, termed iCoagLab, that provides a comprehensive profiling of hemostatic function by measuring activated clotting time (ACT), PT, clot rate, clot stiffness, fibrinolysis, all using the same instrument via inexpensive disposables by evaluating a drop of blood.^38^^,^^41^^,^^43^^,^^44^^,^^46^^,^^64^ The instrument [Fig 4(a)] is a miniaturized and customized embodiment of the general LSR platform, featuring a small laser diode to illuminate the sample and a CMOS camera to acquire the speckle patterns.^43^^,^^44^^,^^46^ In unclotted blood, the continuous motion of light scattering red blood cells and platelets undergoing Brownian motion induces rapid temporal speckle intensity fluctuations. During coagulation, blood viscoelasticity is incrementally modified in accords with key aspects of hemostatic clot formation, including platelet aggregation, fibrin polymerization, cross-linking, stabilization, and eventually fibrinolysis.^41^^,^^84^ The gradual emergence and stabilization of the fibrin–platelet clot with increased modulus restricts the Brownian motion of scattering blood cells, subsequently culminating in reduced optical phase shifts and speckle decorrelation.^38^^,^^41^^,^^43^^,^^44^^,^^46^ Speckle frame series are obtained, every 1 s, during the entire coagulation and several G, i.e., $aG^{*}$, that evolve over-time are evaluated by repeating the processing, as shown in Figs. 4(b) and 4(c). An excellent correspondence is observed between these and the $G^{*}(\omega )$ evaluated using a conventional rheometer during whole blood coagulation [Fig. 4(d)].^41^ To enable comparison with the standard TEG, the modulus, G, is reported at ω=1  Hz [Fig. 4(d)]. The relative change in G, compared to the baseline, unclotted whole blood is output as a graphical profile, along a whole host of coagulation parameters, including the reaction time (R), clot formation time (K), rate of clot formation (α), maximum amplitude (MA), and extent of fibrinolysis (%LY), directly obtained from this profile to assess global hemostatic status.^38^^,^^43^^,^^44^^,^^46^ Utilizing an expanded beam illumination, in conjunction with spatiotemporal analysis of speckle intensity fluctuations further generates the detailed maps of G during coagulation [Fig. 4(e)].^41^

From these maps, incipient microclots are identified at very early times (1 min) in a normal sample.^41^ Nevertheless, in a hypocoagulable specimen, negligible clot formation is observed even after several minutes. These maps suggest that comprehensive coagulation profiling may be achieved at significantly shorter times by monitoring and tracking the rate and the relative strength of microclots in normal and coagulopathic blood specimens.^41^

The unique capability of LSR for visualizing the dynamics of coagulation at the microscale and in real-time provides additional diagnostic information to assess complex blood coagulation deficiencies with high sensitivity at very early stages of coagulation. Successful completion of these pilot studies triggered a series of seminal clinical studies to investigate the LSR in the context of multiple indications at various settings, including core coagulation labs, point-of-care cardiac surgery suites, as well as primary care and in-home settings for anticoagulation monitoring.^38^^,^^43^^,^^44^^,^^46^ The first clinical study compared the LSR with CCT’s and demonstrated a strong correlation between aPTT and functional fibrinogen with ACT (i.e., R+K), and MA, derived from the G-trace, in 50 samples collected from hospitalized patients.^38^ Figure 4(f) displays characteristic G traces for patients’ blood samples with normal (24.9 s) and elevated aPTT (63.5 s). The G trace corresponding to the normal patient maintained a consistently low amplitude during the initial 2 min, followed by a drastic increase in amplitude due to fibrin polymerization and formation of platelet–fibrin network.^38^ In contrast, the G trace corresponding to the patient with elevated aPTT exhibited a significantly prolonged duration of constantly low amplitude, due to delayed fibrin polymerization, caused by deficits of coagulation factors. The curve eventually attained a maximum plateau albeit at a significantly slower rate, after 12 min, suggestive of the diminished rate of fibrin polymerization.^38^ Figure 4(g) shows the representative G-traces of two patients, one with normal and the other with elevated levels of fibrinogen (3.78 and 5.41  g/L, respectively).^38^ A significantly larger MA was noted in the trace corresponding to the patient with high fibrinogen value compared to the normal patient.^38^ The strong correlation between the MA and fibrinogen level was expected since the maximum value of G attained by the clot is related to the strength of the fibrin network, in turn proportional to the fibrinogen content.^38^

The next study investigated the capability of LSR for directing the anticoagulant treatment and hemostasis monitoring in swine blood specimens spiked with varying doses of several common anticoagulants.^44^ Results demonstrated that for all anticoagulants, ACT was prolonged in a dose-dependent pattern and was closely correlated with TEG (r=0.99, p<0.01).^44^ The study also went on to compare the LSR measurements with CCT’s and TEG in patients treated with warfarin with an international normalized ratio (INR: an expression of PT as a ratio) range of 1.0 to 3.6, covering normal as well as the effective therapeutic range for people taking warfarin. A significant correlation was observed between ACT and aPTT (r=0.77, p<0.005). Moreover, LSR and TEG demonstrated a good correlation in terms of MA (r=0.61, p<0.04) and α angle (r=0.63, p<0.03).^44^

Nevertheless, aPTT is not frequently used in the context of monitoring warfarin patients and PT-INR is a more relevant measure of extrinsic coagulation pathways implicated in anticoagulation. Therefore, a separate study was conducted in 60 blood specimens collected from warfarin-treated patients (INR: 0.95–2.71) and blood coagulation was initiated using thromboplastin assay.^44^ These studies demonstrated that the PT-INR measured by LSR significantly correlated with its CCTs counterpart (r=0.94, p<0.001).^44^

Additional studies were conducted to assess the sensitivity, and precision of LSR in quantifying fibrinolysis, through spiking the human blood with a protein involved in the breakdown of fibrin, called tissue plasminogen activator (tPA).^43^ Fibrinolytic activity was assessed by the percentage reduction of MA, termed LY%. The normal controls exhibited a stable LSR amplitude with an MA that was maintained for at least 20 min, whereas tPA-treated samples experienced a severe reduction in MA after only 10 min due to full breakdown of fibrin clot. The LY% measured with both LSR and TEG in 15 patients was strongly correlated (r=0.94, p<0.01).^43^

These studies demonstrate the capacity of LSR for comprehensive coagulation testing, in a multifunctional, handheld instrument. They further highlight the significant clinical impact of the LSR technology in identifying patients at high risk of bleeding or thrombosis, monitoring hemostasis during anticoagulation therapy, and tailoring blood transfusion protocols, to improve patient outcome.^64^

### 4.3 LSR Microscopy and ECM Mechanics

Natural processes such as embryogenesis, organ development, and wound healing are frequently associated with an orchestrated spatiotemporal modulation of the ECM micromechanical properties. On the other hand, aberrant micromechanical remodeling of the ECM gives rise to deadly fibrotic diseases, which account for over 45% of deaths worldwide.^1^ Perturbations to the micromechanical properties of the ECM are also implicated in tumor malignancies.^85^^,^^86^ Therefore, knowledge of the ECM mechanics is crucially important for advancing current understanding of disease etiology and for developing new therapeutic paradigms to counter mechanical aberrations. In this respect, the high-resolution, noninvasive attributes of LSR make it a powerful tool for investigating and addressing key questions concerning the biomechanical mediators of normal development and disease.

Over the years, our group has conducted several studies, demonstrating the capabilities of LSR for quantitative evaluation of the $\left|G^{*}\right|$ at bulk scale in viscoelastic silicone colloids, lipid emulsions, and hydrophobic silicone-based PDMS polymers.^34^^,^^35^^,^^37^^,^^40^ A more recent study detailed and validated the LSR platform for quantifying the frequency-dependent $\left|G^{*}\right|$ of hydrogels over a significantly extended range of moduli, (i.e., 47 Pa to 36 kPa), pertinent to natural and synthetic tissues in normal physiological and pathological states.^42^ Results established the large dynamic range of LSR, by demonstrating the strong correlation with mechanical rheometry (r=0.95, $p<10^{-9}$). The $\left|G^{*}\right|$ values measured by LSR also correlated well with indentation moduli, E, reported by AFM (r=0.92, $p<10^{-7}$), which probe the microscale mechanical properties.^42^ The large dynamic range of LSR encompasses extremely low viscosity biofluids (few mPa) to highly elastic solids (tens of kPa).^34^^,^^42^ The lower limit is set by the frame rate of the camera, and the ability to track the extremely rapid speckle fluctuations is low viscosity liquids.^34^^,^^35^^,^^42^^,^^71^ The upper limit on the other hand is set by the size of scattering particles, a, the ability to resolve their infinitesimal motions, δr, and the thermal energy, $K_{B}T$, to: $K_{B}T/(\delta_{r}^{2}a)$.^42^ The exquisitely sensitive multiple speckle detection offered by the CMOS enables displacements of δr∼Å, leading to an upper limit of tens of kPa.

The significant correlation between LSR and rotational shear rheology is maintained over a range of frequencies. At extremely low frequencies, below 0.01 Hz, the compressional fluctuations exceed the shear counterparts, causing the speckle fluctuations to deviate from the results of shear rheology. On the other hand, the upper frequency limit is set by the inertial effects, which take off as the penetration depth of shear waves ($\propto \frac{1}{\omega }$) becomes comparable to the probe size. In rheometer, this is equivalent to the gap size and the upper limit may be as low as 10 Hz, particularly in low viscosity liquids. In contrast, the submicron size of scattering particles in LSR pushes the upper limit to tens of kHz, opening a new window of frequencies inaccessible to mechanical rheometry. The practical upper, limit however, is further tied to the camera frame rate. By employing acquisition speeds of a few 100 kfps, higher frequencies, in the order of $10^{5}\;\mathrm{Hz}$ may be achieved.

**Fig. 5:**
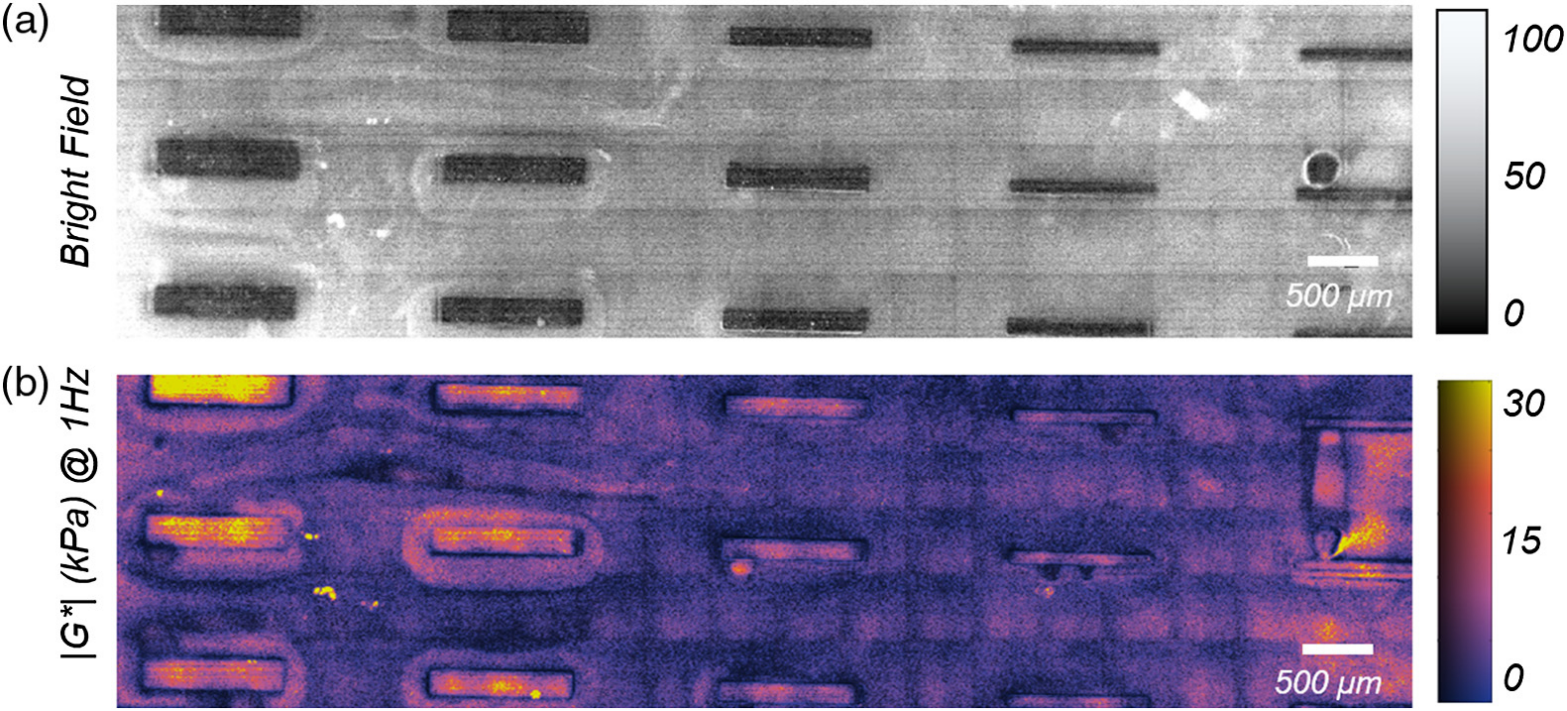
(a) Bright-field image of the microfabricated composite PDMS-PEGDA phantom. A total of 15 PDMS bars are visible within the PEGDA background. The bars in successive columns are 1 mm long and 200, 150, 100, 80, and 60  μm wide, respectively. (b) Spatially resolved $\left|G^{*}\right|$ evaluated at 1 Hz. The color-bar represents the moduli range of 0 Pa to 30 kPa. Elastic bars with thicknesses of a few tens of μm are easily resolved from the background (adapted with modifications from Ref. 42).

**Fig. 6:**
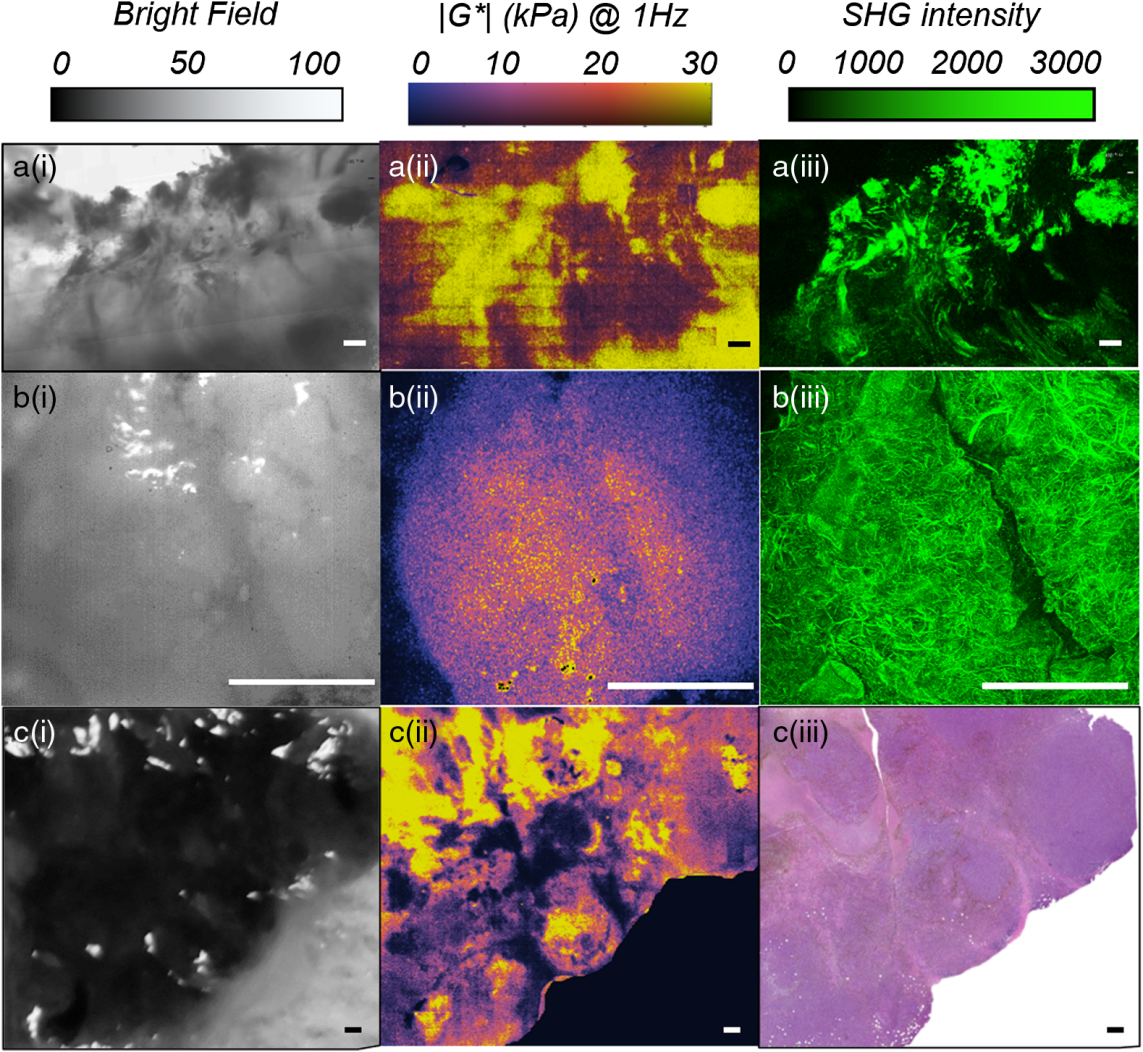
Bright-field images, LSR maps, and either of histology or SHG images of human tissue specimens associated with three different cancers, namely: a(i–iii) breast cancer (invasive ductal carcinoma), b(i–iii) squamous cell lung cancer, and c(i–iii) melanoma skin cancer. Strong qualitative agreement is observed between LSR maps of the viscoelastic modulus and the intuitive perception of stiffness from the histopathological and compositional images in all three cases. Regions of increased viscoelastic modulus, in a–c(ii), coincide with fibrous stroma in H&E section and the foci of collagen accumulation and alignment in the SHG images, in a–c(iii). On the other hand, soft regions of low viscoelastic modulus correspond to cell-dense regions in the H&E section and areas depleted of collagen in the SHG image. The LSR microscopic platform provides the unique capability to map the mechanically heterogeneous tumor microenvironment. Scale bars are 500  μm.

To examine the capability of LSR for offering the combined strengths of standard mechanical rheometry and microindentation, we exploited soft lithography to fabricate a composite PDMS-PEGDA phantoms, featuring stiff PDMS bars of 1 mm long, and 200, 150, 100, 80, and 60  μm wide surrounded by soft PEGDA 5% hydrogel. The substrate was illuminated by an expanded beam, and spatiotemporal processing was employed to obtain the $g_{2}(t)$ curve for individual pixels. Subsequently, the spatially resolved, frequency-dependent 2-D map of $G^{*}$ was deduced, as displayed in Figs. 5(a) and 5(b) for ω=1  Hz. Mechanical rheometry of the constituent materials agreed with $G^{*}$ values reported in the color map for PEGDA 5% (rheometer: 275±122  Pa, LSR: 272±0.9  Pa) and PDMS (rheometer: 10.8±2.1  kPa, LSR: 7±0.03  kPa). The map further emphasized that LSR conveniently resolved features in the order of tens μm. Taken together, these results proved that LSR bridges the advantageous traits of conventional rheometry for measuring frequency-dependent viscoelastic behavior with the opportunity for micromechanical mapping afforded by AFM via a noncontact, all-optical approach. Following phantom studies, the LSR platform has been used extensively in our lab for micromechanical mapping of the tissue microenvironment in different pathological states. Figures 6(ai)–6(iii) display the bright field, LSR map, and the corresponding second harmonic generation (SHG) microscopy image of an invasive ductal carcinoma, a common histological type of breast cancer. The SHG image is provided for the sake of comparison and visualizes the collagen content and architecture. It is to be noted that collagen is a key constituent of the ECM and the main source of tissue tensile strength. Due to traditional lack of high-resolution mechanical imaging tools in the past, cancer mechanobiology researchers have frequently used the architectural features of collagen network, such as density and orientation as proxies to ECM stiffness.^87^^,^^88^ A strong qualitative correlation is observed between the LSR map and SHG image. Close examination of these images reveals that the regions of increased viscoelastic modulus mark the foci of collagen accumulation and alignment. In contrast, pliant areas coincide with fatty adipose tissue. Figures 6(bi)–6(biii) display the bright field, the LSR map, and the SHG image within a lung cancer nodule, over a magnified and significantly smaller region. Like the breast carcinoma above, a close qualitative agreement exists between the LSR map and the SHG image. A diagonal track of decreased stiffness in the LSR map marks a region depleted of collagen, with a similar geometry in the middle of the SHG image. Figures 6(ci)–6(iii) display the bright field, the LSR map, and the hematoxylin and eosin (H&E) of a melanoma skin lesion. From these images, it is appreciated that the soft regions of low viscoelastic modulus in the LSR map correspond to cell-dense regions in the histology image. The stiff regions on the other hand correlate with the dense ECM. The distinct and contrasting micromechanical properties of neoplastic cells and their ECM are likely the key for deciphering the dialogue between the ECM stiffness and intracellular oncogenic signaling. The LSR microscopic platform provides the unique capability to map the mechanically heterogeneous tumor microenvironment. The insight gained from studying these micromechanical features in the context of other hallmarks of cancer will open new paradigms for rheology-informed cancer diagnosis and drug development for improved therapeutic efficacy in the future.

## 5 Summary and Future Opportunities

Mechanical remodeling of the tissue microenvironment is implicated in various stages of human disease, from pathogenesis to progression. The optical mechanical imaging techniques each exhibit exclusive features deemed suitable for probing micromechanical properties at various phases of the disease, in different *in-vitro* and *in-vivo* settings.^13^^,^^20^^–^^22^^,^^27^^–^^33^^,^^38^^,^^41^^–^^46^^,^^49^^–^^51^^,^^57^^–^^62^^,^^64^^,^^86^ Platforms such as Brillouin microscopy, TFM, ERISM, and LSR microscopy are well suited for investigating the micromechanical interactions between cells and their ECM and the skewed intracellular mechanosignaling at the onset of human disease.^13^^,^^26^^,^^29^^,^^42^^,^^57^^–^^60^^,^^86^^,^^89^ By imaging the biomechanical and mechanobiological processes within live cells and ECM, these technologies could improve our understanding of the disease etiology for developing new diagnostic and therapeutic approaches to manage the early stages of disease progression. In this respect, the microscopic LSR platform presents several unique characteristics, including the high spatial resolution, the multifrequency measurement, larger imaging depths (up to 2 mm), and large field of view (several cm), within minutes. Moreover, since LSR does not contact or manipulate the sample during measurement, it offers several distinct advantages for operation in sterile environments where sample contamination by microindentation devices may pose a significant problem. In addition, LSR imaging capability can be easily integrated with other microscopic platforms, to directly interface the micromechanical findings for instance with structural and biochemical context of the cells and their microenvironment. Such an integrated platform provides biologists with a new imaging tool to address key questions concerning the mechanosensitive regulation of cell morphology, physiology, and behavior by ECM components.

In advanced stages of the disease, assessment of the micromechanical properties within the lesions potentially plays a prominent role for improving the diagnosis. Leading examples include the use of intravascular LSR endoscopes for the detection of vulnerable plaques in patients at risk for AMI, intraoperative employment of compression OCE for breast tumor margin assessment, and Brillouin imaging for diagnosis of ocular pathologies.^20^^,^^28^^,^^33^^,^^45^ For true *in-vivo* diagnostic applications, the optical layout often needs to be reconfigured as a compact imaging probe. Active optical elastography techniques face a major challenge in miniaturizing the combined loading and imaging components in a single probe.^10^ On the other hand, due to its passive attributes, LSR does not require a loading mechanism. Therefore, it is elegantly embodied as a miniature endoscope or needle to assess deep tissues and internal organs in a minimally invasive manner. The passive nature of LSR, however, inherently limits this technology to evaluating the linear viscoelastic modulus, which represents the stress–strain ratio under the assumption of low strain level where the relationship is still linear.^72^ While this simplifying assumption is often valid, the complex composition of the tissue could impart a more complex, nonlinear stress–strain relationship, even under physiologically relevant high strain levels.^90^ Future advancements in the LSR techniques could likely enable probing such nonlinear properties, by actively modulating or preloading the tissue with a certain amount of strain to extend the dynamic range of LSR beyond the linear regime.^91^ The current omnidirectional viewing LSR catheter is <1.2  mm in diameter and is comparable in size to commercially available intravascular catheters and considerably smaller than several gastrointestinal endoscopes, opening the opportunity for LSR evaluation in luminal organs via small diameter endoscopes and catheters.^45^ LSR can further be conducted, via a small-bore needle, and effects of pressure gradients during needle insertion may be avoided by delaying speckle image capture by a few seconds following insertion, opening new avenues for imaging in deep inaccessible tissues for research and clinical investigations.^45^

Unlike most optical micromechanical imaging technologies which are still at early stages of clinical development, the LSR-based *in-vitro* diagnostic sensors are at the cusp of maturity on their translational track.^43^^,^^44^ The current LSR-based coagulation sensor is a portable device, with demonstrated diagnostic competency at the point-of-care.^43^^,^^44^ Our next prototype will be a handheld, cell-phone-based, self-testing device, exploiting a battery-operated, small laser diode, and a low-frame rate cell-phone camera, capable of assessing multiple coagulation parameters from a blood volume <40  μl, which enables self-testing via a finger-stick blood draw in the patient’s home.^43^^,^^44^^,^^46^

Despite tremendous developments in optical micromechanical imaging modalities, a common challenge remains the artifacts introduced by physiological motions and vibration noise, which could obscure the readouts of displacement and deformation, as measured in these techniques. The multifrequency nature of LSR measurements along with high frame rate acquisition capabilities enables evaluating sample dynamics over a range of frequencies to isolate passive thermal Brownian dynamics from active displacements occurring at different time scales and loading rates. Another difficulty faced by nearly all these modalities is the ability to reliably quantify the mechanical properties. For instance, in compression OCE, the assumption of mechanically homogeneous tissue, and a uniform stress field is required to translate the deformation to elastic modulus.^10^^,^^17^^,^^18^ Similarly, both transient OCE and Brillouin microscopy make certain assumptions about the tissue density and Poisson ratio to retrieve the modulus from the velocity of acoustic waves.^10^^,^^17^^,^^18^^,^^29^ As elaborated above, knowledge of optical properties and scattering particle size distribution is necessary for LSR to accurately quantify the shear viscoelastic modulus.^35^^,^^37^^,^^40^ We have devised a processing scheme to deduce these parameters from the speckle signal itself.^35^^,^^37^^,^^40^ Nevertheless, our approach for estimating the scattering particle size, a, is currently limited to a: 100 nm to 3  μm.^40^ We are currently investigating additional processing steps to extend this range. The limited penetration depth and axial resolution are also a common issue among different optical micromechanical imaging techniques. In OCE techniques, the penetration depth is often limited to a few mm, set by the penetration depth of the underlying OCT system. On the other hand, the axial resolution could be significantly less than the OCT, particularly in compression OCE, due to the need for calculating the gradient of displacements.^17^ In the case of Brillion microscopy, confocal acquisition severely restricts the imaging, while enabling a superior axial resolution.^26^^–^^29^^,^^49^^–^^51^ As mentioned earlier, due to the limitations in evaluating out-of-plane displacements, TFM often exhibits a poor axial resolution.^59^^,^^60^ In the case of LSR, acquisition of diffuse multiply-scattered light extends the penetration depth to over 10 times the mean free path of photons (i.e., $10\times l^{*}$) corresponding to tens of millimeters in biological tissue. Nevertheless, due to absence of a coherence gating mechanism, the measurements are typically depth-integrated. Fundamental mechanobiology studies have emphasized the importance of three-dimensional (3-D) micromechanical context in mediating the disease. In addition, clinical findings stress the rich diagnostic features appreciated from volumetric imaging in 3-D that may be obscured in 2-D projections. We have previously exploited spatiotemporal processing of speckle images to measure and map the depth-resolved mechanical properties in atherosclerotic plaques in 3-D and identify rupture-prone lesions.^31^^,^^61^ These efforts pave the path for the development of next-generation LSR technologies featuring depth-resolved volumetric imaging capabilities.
